# Metagenomic analysis of microbiome spatial dynamics in urban river confluence affected by city wastewater

**DOI:** 10.1186/s44342-025-00054-3

**Published:** 2025-12-04

**Authors:** Nahid Parwin, Sangita Dixit, Sriansh Das, Rajesh Kumar Sahoo, Enketeswara Subudhi

**Affiliations:** 1 Centre for Biotechnology, School of Pharmaceutical Sciences, Siksha ‘O’ Anusandhan (deemed to be University), Kalinga Nagar, Ghatikia, Bhubaneswar, Odisha 751003 India; 2 DBT-Institute of Life Sciences (DBT-ILS), Bhubaneswar, Odisha India

**Keywords:** Kathajodi, Pollution, Anthropogenic activities, Metagenomic, Bacterial diversity, Functional profiling

## Abstract

**Background:**

Environmental pollutants have a profound impact on microbial dynamics. This study highlights the influence of anthropogenic activity on the shift in bacterial diversity in the catchment area compared to upstream and downstream at Kathajodi, using a metagenomic approach for the first time in River Kathajodi.

**Methods:**

Water samples were collected from upstream, catchment, and downstream locations and transported at 4°C to the laboratory for DNA extraction, library preparation, sequencing, and physicochemical analysis employing inductively coupled plasma. The extracted DNA was sequenced via the Illumina HiSeq platform and analyzed through MG-RAST for taxonomic and functional classification using KEGG and COG annotations. Statistical diversity analysis, including rarefaction curves, alpha- and beta-diversity indices, and Venn diagrams, provided insights into microbial composition and community variations across sites.

**Results:**

A significant abundance of pollution indicator members of phylum *Bacteroidetes* (29.82%) in the catchment (CM), highly contaminated with metals, fecal, and other organic pollutants, could be attributed to their high metabolic capabilities to degrade them. The pristine upstream (US) exhibited an abundance of *Shewanella* (25.04%), *Pseudomonas* (17.35%), and *Synechococcus* (5.62%). The CM, influenced by high anthropogenic activity, showed higher abundances of *Flavobacterium* (5.20%), *Arcobacter* (4.05%), and *Bacteroides* (3.88%). In contrast, downstream (DS), with fewer anthropogenic activities, displayed higher abundances of *Aeromonas* (4.40%), *Acidovorax* (0.52%), and *Acidimicrobium* (0.32%). The highest bacterial diversity of CM could be due to the influence of the physicochemical properties of city waste effluent. From the Venn diagram, 73 common OTUs at the genera level were observed in all three sites, which indicates that the native microflora of the river water niche remains unaffected irrespective of the temporary changes in the vicinity. The functional profiling through KEGG and COG revealed that CM was enriched in carbohydrate metabolism (12.11%), while DS exhibited higher contributions to amino acid metabolism, along with the highest relative abundance of general function prediction (R) (12.89%), all indicative of stress adaptation and metabolic flexibility under polluted conditions. The clean upstream is home to oxygen-loving helpful bacteria, the catchment supports nutrient-hungry and sewage-linked microbes, while the downstream is dominated by metal-tolerant and possibly harmful bacteria, showing the clear impact of human activities along the river.

**Conclusions:**

The marked shift in bacterial diversity between US, CM, and DS regions highlights the ecological consequences of anthropogenic impact. These findings emphasize the need for effective environmental management to safeguard water quality and prevent undesirable health issues.

**Supplementary Information:**

The online version contains supplementary material available at 10.1186/s44342-025-00054-3.

## Background

The rapid growth in human population, industrialization, and urbanization has significantly increased pollution levels in freshwater ecosystems, particularly rivers. Rivers, though, play a critical role in sustaining human and ecological needs, yet they get severely deteriorated. A wide array of untreated pollutants, including fecal waste, industrial effluents, oils, plastics, pesticides, and heavy metals, is discharged into rivers, leading many to resemble open drains rather than the lifelines for ecosystems [1]. This alarming trend poses severe threats to aquatic ecosystems and human health on a global scale [1] and is particularly evident in India, where major rivers face severe pollution levels, affecting both human populations and the surrounding environment [2–4] due to unregulated wastewater discharge [3]. The consequences of such pollution include shifts in microbial diversity, propagation of antimicrobial resistance (AMR), and diminished ecosystem health [3, 5].

While many studies focus on water quality, despite growing recognition of these issues, significant knowledge gaps persist, paying less attention to the microbial quality of rivers in developing countries [6]. These research gaps are compounded by inadequate wastewater treatment facilities, particularly in urban areas, where growing populations exert immense pressure on basic services such as water supply and waste management [6]. Prokaryotes, with their metabolic adaptability, can utilize pollutants as growth substrates and predominate, but these adaptations may exacerbate ecological imbalances and health risks [3].

Traditional culture-based characterization, commonly employed for microbial studies, is labor-intensive but limited to the cultivable fraction of the microbial communities [6]. In contrast, metagenomics, which employs next-generation sequencing technologies, enables comprehensive analysis of microbial communities and their functional potential, offering deeper insights into the roles of microorganisms in polluted environments [3].

The Kathajodi River, a tributary of the Mahanadi River in Cuttack, Odisha, exemplifies the challenges faced by urban rivers in India. Cuttack, with a population exceeding 600,000, generates approximately 65.44 million liters of wastewater daily, most of which is discharged untreated into the river [7]. This has transmuted the Kathajodi River into a heavily polluted water body, unsuitable for human consumption and designated as a level C pollution zone by the Odisha State Pollution Control Board [8]. Previous studies have reported the presence of heavy metals such as lead, copper, and chromium, along with drug-resistant bacteria, including *Klebsiella pneumoniae* and *Acinetobacter baumannii*, in the river [9, 10].

Although the Kathajodi River receives large volumes of untreated municipal and industrial effluents, little is known about how these inputs alter its microbial communities across different sites. Previous work on this river has focused mainly on physicochemical parameters and metal loads, but the microbiome response has not been systematically explored. In particular, three important questions remain unanswered. First, how does microbial diversity vary between the relatively pristine upstream, the wastewater-dominated catchment, and the downstream stretch? Second, which bacterial groups act as indicators of local pollution pressures, including fecal and heavy metal contamination? Third, how do functional gene profiles shift in response to the gradients of organic load and metals recorded in the system?

To address these questions, we used Illumina-based high-throughput metagenomic sequencing on water samples collected from the upstream, catchment, and downstream regions of the Kathajodi River. The approach allowed us to connect microbial diversity and functional attributes with the distinct pollution signatures observed at each site. This work provides the first spatially resolved account of microbiome dynamics in the Kathajodi, an urban river confluence under heavy anthropogenic pressure. By treating the river as a case study, we show how human activities shape microbial communities and their functions. The findings also highlight broader implications for public health and water quality management, as pollution-driven microbial shifts may alter ecosystem resilience and pathogen risks.

## Material and methods

### Study area and sample collection

Cuttack, the largest urban center in the Cuttack district, is situated between latitudes 20°03″ and 20°40″ North and longitudes 84°58″ and 86°20″ East. Recognized as the cultural hub of Odisha, it lies at the confluence of the Mahanadi and Kathajodi rivers. The city experiences a tropical climate, with summer temperatures often exceeding 40 °C and winter temperatures dropping to around 10 °C. The average annual rainfall is approximately 1892.55 mm, largely driven by the South-West monsoon. While 76% of the population relies on agriculture, the city also supports various small-scale industries, including handicrafts, cottage industries, and processing units focused on chemicals, textiles, and leather products [11].

**Fig. 1:**
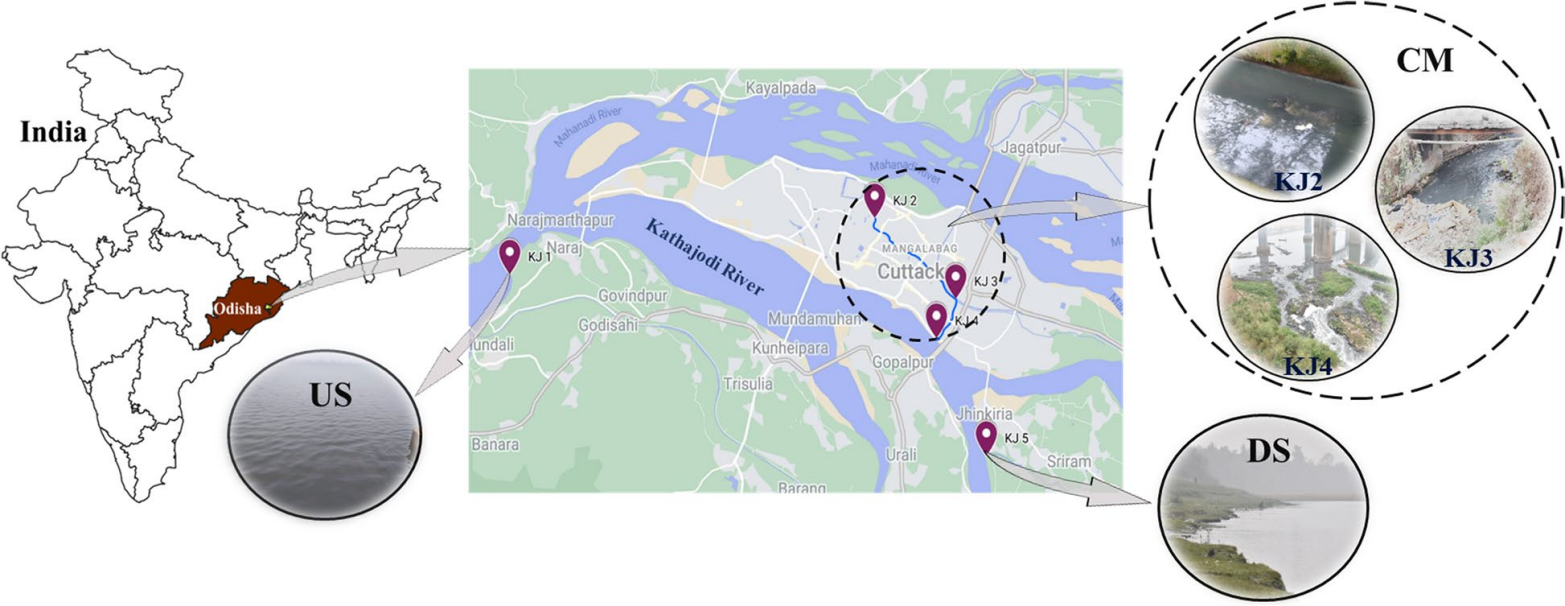
The map shows the Cuttack city adjoining study areas at KJ1—US of the Kathajodi River, 12.3 km ahead the city; KJ2 and KJ3—municipal drainage system within the city, with the extreme amount of anthropogenic activity; KJ4—the confluence where the river and the city’s drainage meet, and upon pooling the samples from these three sites, it is represented as catchment (CM); and KJ5 3.4 km from downstream of the river away from the city. The map was constructed using QGIS 3.34.0 software

In March 2020, water samples were collected from three key locations along the Kathajodi River for analysis. The sites included the following: an upstream (US) point located 12.3 km from the city (20°27′40.7″ N; 85°45′21.1″ E), the catchment area (CM) comprising wastewater discharge points (KJ2—20°28′34.7″ N; 85°52′16.9″ E, KJ3—20°27′16.8″ N; 85°53′49.4″ E, and KJ4—20°26′39.1″ N; 85°53′27.4″ E), and a downstream (DS) point 3.47 km from the city (20°24′46.1″ N; 85°54′24.2″ E) (Fig. 1). To ensure homogeneity, three samples were taken from each site at intervals of 100 m and at a depth of 0.5 m from the surface. These were pooled to create a composite sample representing each location.

Samples were collected in sterilized, amber-colored 500-mL bottles, appropriately labelled, and stored in ice boxes to maintain a temperature of 4 °C during transportation to the laboratory. This controlled storage ensured the integrity of the samples for subsequent analysis. Physicochemical parameters were measured at the State Water Testing Laboratory, Odisha, using inductively coupled plasma mass spectroscopy (ICP-MS; NexION 200). Certified reference materials (CRMs) from Perkin Elmer TruQms (lot numbers CL13-93HGY1 for Hg and CL4-35MJY1 for other metals) were used during the analysis. The results were interpreted according to guidelines provided by the United States Environmental Protection Agency [12] and the World Health Organization [13].

### Extraction of DNA, library preparation, and sequencing

The entire composite water samples (1.5 L) were filtered using a (0.22 μm) Nylon Membrane filter (HiMedia). Then, the membrane was proceeded with for the extraction of DNA using the DNeasy PowerWater Kit (QIAGEN) as per the manufacturer’s protocol. The quality of the extracted DNA was evaluated using 1% agarose gels and was quantified using a NanoDrop Lite Spectrophotometer (Thermo Fisher Scientific, MA, USA). The DNA sample was then stored at −20 °C for further analysis.

The metagenomic sequencing was conducted by Agrigenome Labs Pvt. Ltd. in Kerala, India. Initial libraries were constructed using the Nextera XT DNA Library Preparation Kit from Illumina Inc., USA, and were subsequently diluted to a concentration of 10.0 pM. The library size distribution was assessed using an Agilent 2100 Bioanalyzer (Agilent Technologies, USA). Sequencing was performed using the Illumina HiSeq ×10 for 2 × 150-bp paired-end read chemistry with the Cluster Kit v4 (San Diego, USA). The raw metagenomic sequence data have been submitted to the NCBI’s Sequence Read Archive (SRA) database under the BioProject (PRJNA1008397), with accession numbers SRR25894123 (DS), SRR25894124 (CM), and SRR25894125 (US).

### Bioinformatic analysis

Initially, adapter and primer sequences from amplicons were eliminated using Cutadapt. Subsequently, we removed reads of short length (< 75 bp) and those with low quality using Trimmomatic version 0.40. The quality-filtered forward and reverse reads were then submitted to the Metagenomic Rapid Annotations using the subsystems Technology (MG-RAST) server. To ensure data integrity, all sequences underwent demultiplexing and paired-end joining. Subsequently, paired-end sequences were pre-processed, and low-quality reads were trimmed, with adapter removal and selection of trimming thresholds carried out using SolexaQA. We implemented the DRISEE (Duplicate Read Inferred Sequencing Error Estimation) technique to remove any artificially duplicated reads from the initial sequence dataset. To identify ribosomal DNA, we employed SSU databases with a 97% identity cluster, utilizing VSEARCH against a curated RNA database. Subsequently, sequence reads were clustered at a 97% identity threshold using cd-hit. The BLAST tool was then applied to identify the best-hit classification at a 97% identity threshold, employing a 1e-06 *E*-value and requiring a minimum alignment length of 50 base pairs. The taxonomic profiling, encompassing phyla, classes, orders, families, genera, and species, was comprehensively annotated. For functional gene annotation, we applied hierarchical classification with an *E*-value cutoff of 1e-06, based on the SEED subsystems with KEGG and COG classification of the MG-RAST, following the approach outlined by [14].

### Statistical diversity analysis

A rarefaction curve was generated to investigate the species richness of the overall microbial community via the Phyloseq in R. The microbiomeSeq R package was employed to estimate alpha diversity (Chao1, Shannon, Simpson, and Observed) between the samples. To illustrate the number of common and unique operational taxonomic units (OTUs) at the genus level among three distinct sample sites, a Venn diagram was generated using the online tool Venny [15]. Past CCA statistical approaches were utilized to examine correlations between environmental parameters and the top 30 genera (PAST: Paleontological Statistics Software Package for Education and Data Analysis) [16].

## Result and discussion

### Analysis of physiochemical parameters

Our previous reports on the Kathajodi River, where we have analyzed the physiochemical parameters and metal concentrations across US, CM, and DS regions, reveal significant spatial variability in pollution levels, with specific parameters serving as indicators of distinct pollution sources (Table S1). The upstream region was relatively pristine, whereas higher pollution loads were observed in the catchment and downstream regions [9].

In the US area, water quality parameters such as biochemical oxygen demand (BOD), total dissolved solids (TDS), and electrical conductivity (EC) are well within the limits recommended by WHO, indicating minimal anthropogenic influence. The BOD level is 4330 µg/L, below the WHO threshold of 5000 µg/L, suggesting low organic pollution. The lower levels of TDS and EC (120,000 µg/L and 185 µS/cm, respectively) reflect limited ionic load and minimal contaminant intrusion, which can be attributed to the absence of industrial or agricultural activities and the natural, undisturbed state of the upstream environment [17]. Metals such as iron (105 µg/L), manganese (8.08 µg/L), and cadmium (0.002 µg/L) are also present in concentrations below WHO limits, further supporting the unpolluted status of this region.

In contrast, the CM area shows alarmingly high pollution levels, with BOD rising to 11,700 µg/L, more than twice the acceptable limit, and TDS spiking to 272,000 µg/L. The elevated EC value of 419.03 µS/cm indicates a substantial ionic load. High BOD values point to organic pollution from untreated sewage and industrial effluents [18]. Elevated TDS and EC are indicative of dissolved ions from fertilizers, detergents, and salts from industrial discharges [19]. Previous studies also revealed that the sites highly impacted by anthropogenic activity led to high salinity, TDS, and EC levels and were declared unsuitable for irrigation [20]. Metals such as iron (1190 µg/L), manganese (401 µg/L), cadmium (5.03 µg/L), and aluminum (779 µg/L) exceed WHO limits, underscoring the impact from mining activities, electroplating effluents, and agrochemical use in this region [21, 22]. The Fe concentration (1190 µg/L) in water samples was found to be exceptionally higher, possibly due to the direct discharge of various effluents from textile, milling, plating, and surface finishing industries surrounding the city. It promotes the absorption of other toxic metals such as Cu and Pb [23].

Downstream (DS) water quality shows slight improvement, with BOD decreasing to 5660 µg/L and TDS reducing to 152,000 µg/L. This reduction is likely due to natural attenuation mechanisms such as dilution and sedimentation [24]. However, metal concentrations remain a concern for iron (920 µg/L) and aluminum (715 µg/L), being still above WHO limits. Elevated levels of arsenic, mercury, beryllium, and cobalt downstream suggest sediment transport and remobilization of contaminants, as well as contributions from point sources like wastewater treatment plants (WWTPs) and agricultural runoff [25]. The persistence of these pollutants downstream is also influenced by chemical transformations and anoxic conditions in the river ecosystem.

The presence of specific physicochemical parameters is indicative of various sources of pollution. High BOD values point to organic pollution from untreated sewage and industrial effluents [18]. Elevated TDS and EC are indicative of dissolved ions from fertilizers, detergents, and salts from industrial discharges [19]. Metals such as cadmium and aluminum often originate from mining activities, electroplating, and fertilizers, while lead and zinc may stem from vehicular emissions and urban runoff [26, 27].

The higher concentrations of chromium and silver observed in the upstream region could be attributed to atmospheric deposition, while sodium and potassium levels in the catchment area reflect localized pollution from agricultural and domestic sources. Downstream pollution by arsenic and mercury may arise from sediment transport and point sources such as WWTPs or industrial operations along the river course [25, 28].

Heavy metals exert long-term selective pressure on bacteria due to their persistence in the natural environment and resistance to degradation [29, 30]. Consequently, bacteria develop molecular strategies to resist heavy metals and antibiotics, often utilizing shared genetic mechanisms [31]. In surveillance of the Mahanadi River, it has been observed that its water quality is deteriorating significantly as it flows downstream. This deterioration is primarily driven by factors such as increased nutrient input, fertilizer runoff, pollutant dissolution, human activities, and the prolonged journey of surface water before reaching downstream areas [32].

### Characteristics of metagenomic datasets and biodiversity indices

Approximately, 24.53-gb paired-end metagenomics sequences were obtained for the three different locations together. A total of 15,522,929,252 bps sequences were generated after pre-processing (merge, quality filter, and duplicate removal) of the reads with a mean GC percent of 52 ± 12.66 (Table S2). Clustering of reads at a 97% similarity threshold produced a total of 1928 OTUs after filtering out singletons, which were broadly classified into bacteria, eukaryotes, archaea, viruses, and a small fraction of unclassified sequences. Bacteria clearly dominated across all sites (81.9–97.9%), with much smaller contributions from eukaryotes (1.9–11.9%), archaea (up to 0.6%), and viruses (up to 1.6%) (Table S3A). Diversity estimates further underscored strong site-specific contrasts. The catchment site (CM) stood out with the highest observed richness (1475 OTUs), supported by a Chao1 estimate of 2106, and also showed greater evenness (Shannon 4.13, Simpson 0.88). US communities were moderately diverse (881 OTUs, Chao1 1465; Shannon 3.15; Simpson 0.85), while DS samples were strikingly depauperate, with only 160 OTUs observed, a Chao1 of 244, and substantially lower diversity indices (Shannon 1.67; Simpson 0.64) (Table 1).

**Table 1:** Estimation of alpha index of bacterial microbiomes

Sites	Observed	Chao1	Shannon	Simpson
US	881	1464.52	3.15	0.85
CM	1475	2106.62	4.13	0.88
DS	106	244.00	1.67	0.64

**Fig. 2:**
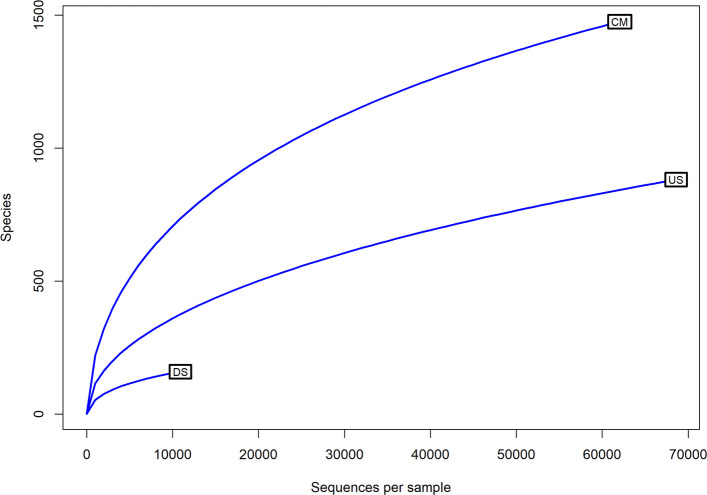
A rarefaction curve illustrates the species richness of individual metagenome samples identified in shotgun metagenome datasets, with each curve corresponding to a specific sample site. The numbers in parentheses accompanying the curve labels indicate the species richness found in each metagenome. The *x*-axis denotes the sequence per samples, while the *y*-axis represents species counts

A rarefaction curve was generated to evaluate the authenticity and adequacy of sequencing in capturing microbial diversity across sites [33]. Rarefaction is commonly employed in microbial ecology to standardize sequencing depth and to visualize whether the observed taxon richness reflects true community composition or is influenced by sampling effort. In this study, the curves revealed distinct differences among sites [34]. The CM showed the steepest rise and highest richness, indicating both sufficient sequencing coverage and a more diverse community likely shaped by mixed anthropogenic inputs. The upstream site (US) displayed intermediate richness, consistent with relatively lower disturbance, while the downstream site (DS) exhibited an early plateau and the lowest richness, suggesting that sequencing depth was adequate to capture most taxa present but that overall diversity was reduced under strong pollution-driven selective pressures. These patterns confirm that the sequencing effort was robust across samples, and that the observed richness differences are ecological in nature rather than artifacts of unequal coverage (Fig. 2).

### Comparative structural diversity of bacterial communities

**Fig. 3:**
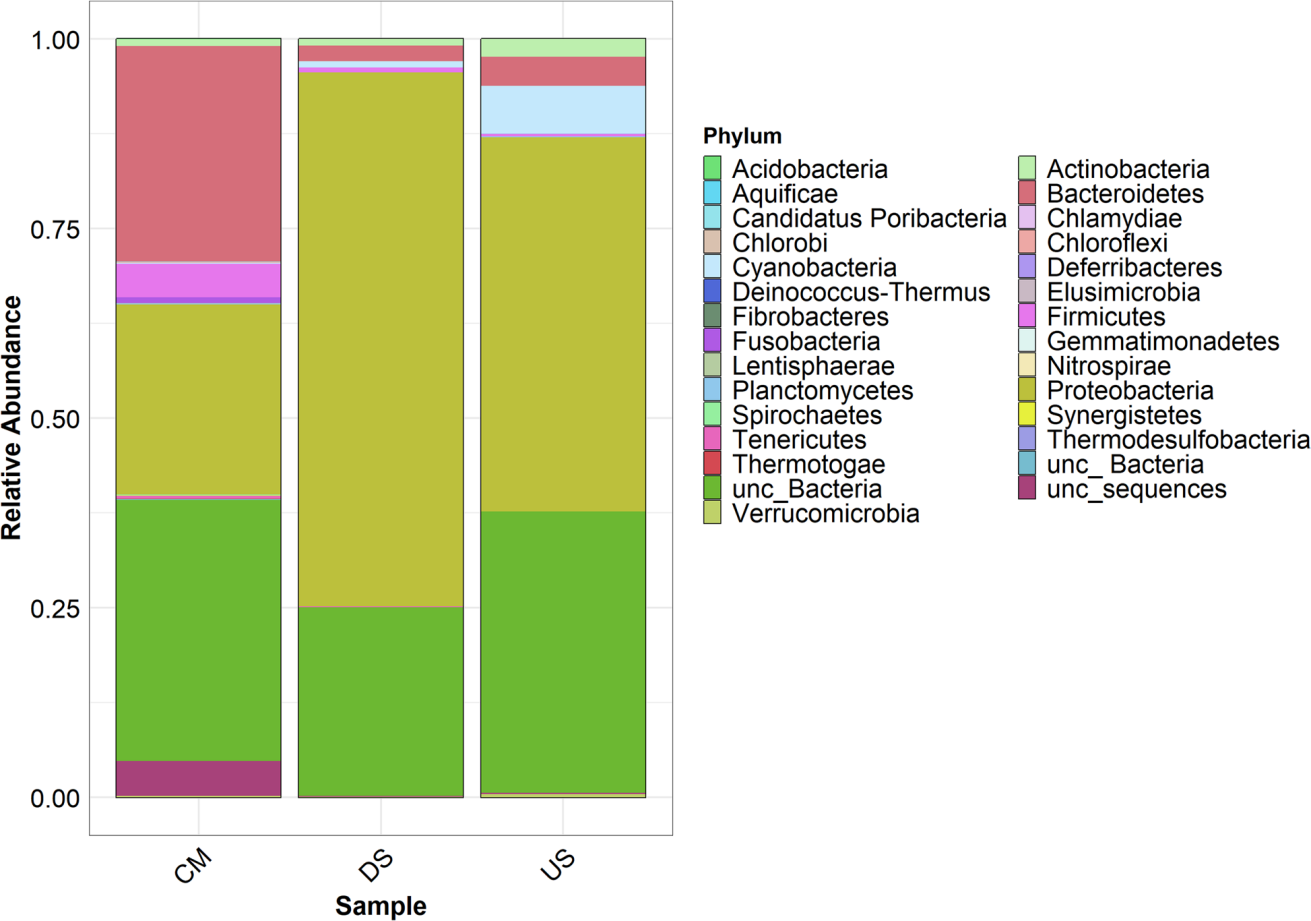
The stacked bar plot illustrates the proportional distribution of bacterial operational taxonomic units (OTUs) classified at the phylum level across individual samples

The comparative taxonomic analysis classified three samples with a range variation from 25 to 26 numbers of phyla, 24 to 49 classes, 53 to 105 orders, 79 to 226 families, 106 to 593 genera, and 881 to 1475 species (Table S2). *Proteobacteria* emerged as the most dominant phylum across all regions, with relative abundances of 70.413% in DS, 49.318% in the US, and 25.063% in CM (Fig. 3). Their dominance can be attributed to their metabolic versatility and resilience to environmental stresses, including heavy metal contamination and organic pollution [35–37]. *Proteobacteria* have significant tolerance to heavy metals such as Zn, Pb, and Cd, as reported in previous studies [38]. The presence of metals like Pb, Zn, and Cd, alongside elevated BOD, COD, and EC levels observed in previous studies [9], supports their prevalence, particularly in DS and CM regions

The higher abundance of *Proteobacteria* in DS suggests a stable microbial community adapted to the long-term presence of pollutants. In CM, the relatively lower abundance of *Proteobacteria* may reflect competitive pressures and the influence of untreated sewage favoring other phyla like *Bacteroidetes*. In the US, *Proteobacteria* benefit from cleaner conditions, contributing to biogeochemical cycling processes [39].

*Bacteroidetes* were most abundant in CM (29.450%), followed by US (3.837%) and DS (2.034%) (Fig. 3). Their dominance in CM is indicative of fecal contamination from untreated sewage and agricultural runoff, consistent with their role as markers for fecal pollution due to their prevalence in the intestines of warm-blooded animals [40]. The correlation of *Bacteroidetes* with TSS, EC, BOD, and COD, and their negative relationship with DO, explains their minimal presence in the US and reduced abundance in DS, where dilution and competition with *Proteobacteria* occur [40, 41]. The detection of *Bacteroidetes* in polluted waters highlights chronic sewage pollution, as reported in studies from various global contexts [42].

*Cyanobacteria* exhibited the highest abundance in the relatively pristine US region (6.333%) compared to CM (0.089%) and DS (0.824%) (Fig. 3). In the US, favorable environmental conditions like nutrient availability, water depth, and optimal temperatures promote their growth. However, their abundance declines significantly in polluted CM and DS regions due to the toxic effects of heavy metals like As, Cr, and Cd, which inhibit chlorophyll synthesis and disrupt photosynthetic processes [43–45]. Fe, Al, Ba, Zn, Cu, and Mn at lower concentrations promote the abundance, but with increasing concentration, the chlorophyll synthesis of *cyanobacteria* is inhibited [41, 46]. Different heavy metals have varying effects on growth rates [47]. Additionally, the physiological stress caused by these metals can lead to altered protein profiles and decreased photosynthetic efficiency, further reducing their abundance [48]. Other factors, such as high concentrations of nutrients leading to eutrophication and altered water flow dynamics, can also contribute to the lower abundance of *cyanobacteria* in polluted waters [49]. However, the highest number of unclassified bacteria was observed phylum level in the US (37.081%) sample, followed by CM (34.585%) and DS (24.879%) (Table S3B).

### Anthropogenic impact on genera diversity shift

**Fig. 4:**
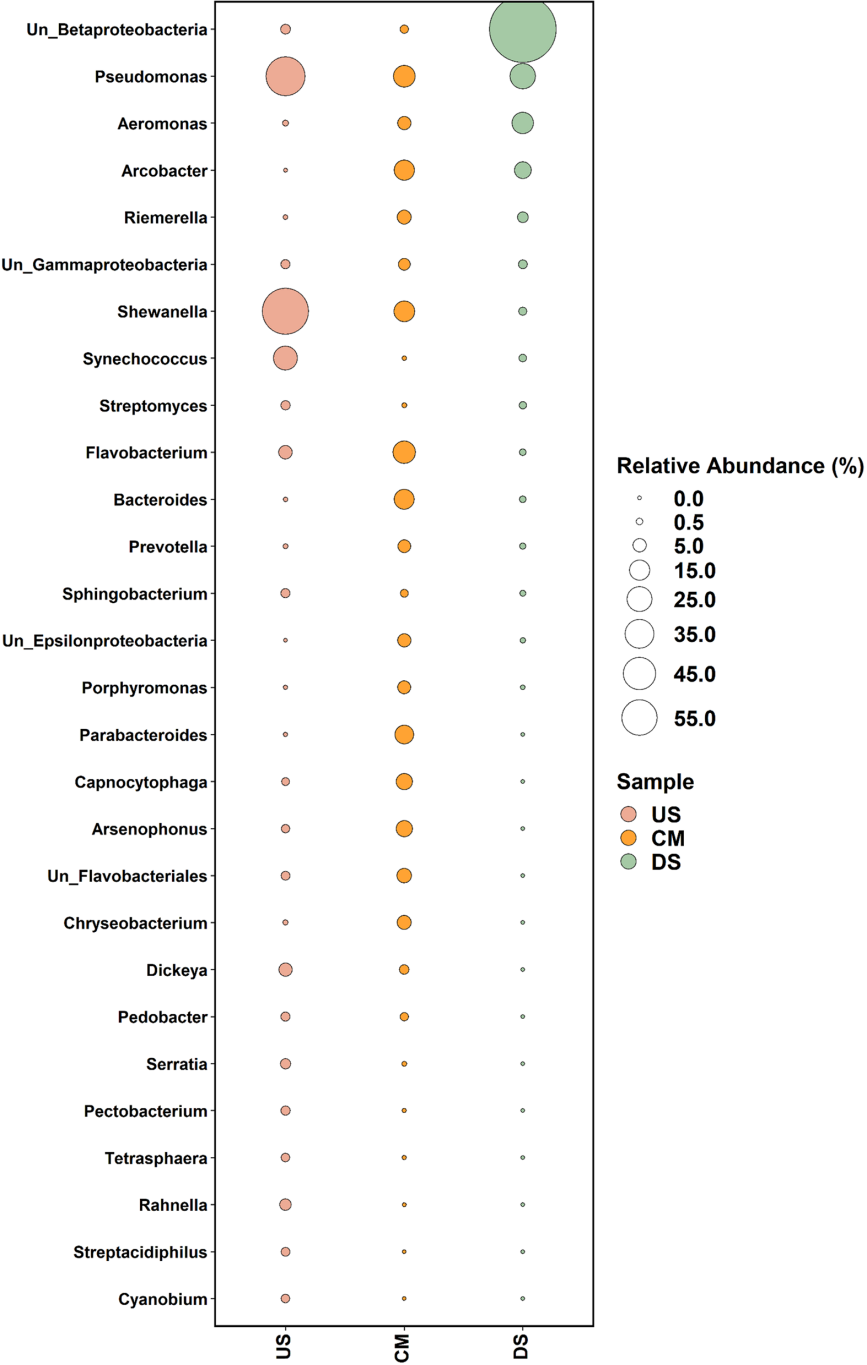
A bubble plot illustrates the top 20 microbial compositions at the genus level, where the circle size corresponds to the relative abundance of a specific genus (%) determined through metagenomic mapping. The color of each bubble indicates its origin from the respective samples

The majority of genera were identified predominantly in the CM (190), followed by the US (104) and DS (26) (Table S3F). The US region of the river, situated before the city and characterized by its pristine nature with minimal anthropogenic activities and abundant natural flora, exhibited the highest number of *Shewanella*, *Pseudomonas*, and *Synechococcus*, compared to DS and CM at the genus level (Fig. 4). *Shewanella* with 25.806% was abundant in the US, followed by 4.030% and 0.266% abundance in CM and DS, respectively. It is more abundant in the US due to higher organic matter, total nitrogen, and carbon presence, which promote its growth [50]. US conditions are more favorable, while DS areas have lower oxygen and diluted nutrients, limiting *Shewanella*’s abundance [51]. The least abundance in CM may be due to the presence of metals like Hg and Cd and a high concentration of Fe–Mn that produces ROS, which disrupts the cell wall and may interfere with the key metabolic processes including respiration, metal reduction, and denitrification pathways, ultimately leading to cell death [51–54]. Polluted waters foster competition with other bacteria, reducing *Shewanella*’s abundance [55]. With 17.190% abundance, *Pseudomonas* was the second most abundant in the US among the three locations, followed by 6.439% in DS and 4.510% in CM (Fig. 4).

*Pseudomonas* species, particularly *Pseudomonas aeruginosa*, are prevalent in clean river water due to their adaptability to various environments, including oligotrophic conditions, and their tendency to colonize biofilms on surfaces like plumbing fixtures [56]. These bacteria are commonly found in freshwater and are associated with human disease, thriving in natural waters such as lakes and rivers [57]. The abundance of the natural flora in the US region leads to the abundance of some species of *Pseudomonas* that have been identified to promote plant growth and suppress pathogens, making them valuable for biological control [58]. *Pseudomonas* efficiently decomposes organic substances in aerobic conditions found in clean water, and their abundance is influenced by environmental factors such as total nitrogen (TN), total phosphorus (TP), total organic carbon (TOC), and the presence of metals like Cu, Zn, and Cd [59]. Metals like Cd, Cu, Ni, Cr, Zn, and Pb at lower concentrations are known to increase *Pseudomonas* abundance, whereas the same metals at higher concentrations especially Hg and Ag at lower concentrations inhibit its growth or can block the transmembrane transport channels and disrupt cell wall integrity, killing the bacteria [60–63]. The higher abundance of *Pseudomonas* in US areas, compared to DS and polluted CM zones, is due to the reduced influence of anthropogenic activities and pollutants in the US, providing a cleaner environment that supports their growth [59]. Pollution from industrial and agricultural sources in DS increases nutrient loads and the presence of toxic metals, which negatively impacts *Pseudomonas* and favors other bacterial species [64]. Similar patterns have been observed in the Yamuna River in India, where *Pseudomonas* species were found to be more abundant in less polluted upstream regions compared to heavily contaminated downstream areas and industrial catchments [65].

*Synechococcus* (5.569%), a freshwater *cyanobacterium*, is significantly more abundant in unpolluted US river areas compared to DS (0.008%) and polluted CM (0.211%) regions (Fig. 4). This pattern is attributed to several environmental and anthropogenic factors. Research shows that in pristine US areas, *Synechococcus* benefits from oligotrophic conditions, lower levels of heavy metals, and minimal human impact [66]. *Synechococcus* is also known for its prolific contribution to oxygen production in both freshwater and marine ecosystems and thrives in various environments, which may also be a cause of high DO in this region as compared to the other two study areas [67]. In contrast, cleaner upstream areas showed reduced *Synechococcus* abundance, which might be due to slight increases in nutrient levels and minor anthropogenic influences. Polluted CM areas exhibit negligible *Synechococcus* presence, as reports have suggested that the presence of heavy metals significantly affects its populations and the presence of heavy metals contamination by elements like Cd, Pb, Co, As, Cr, and Ni, alongside increased organic pollutants in river Kathajodi, may be the cause of their reducing abundance [68, 69]. On the contrary, metals like Fe, Mn, and Zn can enhance *Synechococcus* growth at lower concentrations [70]. Anthropogenic activities, including agricultural runoff, industrial discharge, and urbanization, increase pollution downstream, leading to higher levels of contaminants and reduced *Synechococcus* populations [64]. Other environmental factors also play a role. *Synechococcus* thrives in higher pH levels and areas with clear water, conditions more prevalent in less polluted upstream sections [71]. Low total nitrogen and total phosphorus levels at upstream create favorable conditions for *Synechococcus*, as they prefer oligotrophic environments with fewer nutrients [72]. Similar patterns are observed in other river systems. In the Yangtze River, *Synechococcus* is more abundant in unpolluted upstream areas, while downstream and polluted catchment regions show significant declines [73]. Comparable trends have been noted in the Mississippi River, where *Synechococcus* thrives in upstream areas and declines in polluted downstream regions [74].

On the other hand, the CM area is influenced by high human activity. The municipal drainage system serves as the primary conduit for disposing of waste generated by the city’s populace, such as agricultural runoff, industrial wastewater, traffic pollution, vehicle exhaust, and biomedical waste from major healthcare facilities [27, 28, 75] which later converge to the river at the CM site. The following genera showed relatively increased abundances: *Flavobacterium*, *Arcobacter*, and *Bacteroides* at this site of the study as compared to the other two (Table S3F). *Flavobacterium* dominated the catchment area with a significant abundance of 4.875%. However, this genus’s presence was less, with only 1.337% in upstream and a mere 0.128% as the water moved downstream (Fig. 4). *Flavobacterium*, being a bioindicator of pollution, fecal contamination, and a pollution-tolerant genus, is significantly more abundant in the polluted catchment areas, as seen in research conducted on the Chattahoochee River in the USA and Olifants River catchment in South Africa [76, 77]. It dominated the catchment area due to heavy metal presence (Pb, Ni, Cd, Zn, Co, Al) and multifaceted pollution from industrial and agricultural activities, which create conditions favoring its growth [38, 77, 78]. In contrast, upstream sites had moderate *Flavobacterium* abundance due to lower pollution levels and cleaner water [79]. At downstream, the abundance further declined, likely due to the presence of other competing bacteria like *Proteobacteria*, favored by fecal and organic pollution and dilution of pollutants [40, 41, 66, 80]. A study in the Jialing River noted an upward trend in taxonomic clusters of potential fecal indicator bacteria, including *Flavobacteria* and *Bacteroidia*, in urban water [81]. Another study in the Wonderfonteinspruit catchment area, in South Africa, showed a significant abundance of *Flavobacteria* due to anthropogenic activities [82]. It has been reported that the members of the genus *Flavobacterium* are known for their widespread presence in natural environments, primarily inhabiting aquatic ecosystems that span a spectrum of salinity levels, encompassing both freshwater and seawater environments [83]. *Flavobacterium*, being a highly opportunistic pathogen, primarily affects aquatic species; its potential to cause severe diseases in humans cannot be overlooked. It causes *Flavobacteriosis* in fish and humans; it is known to cause meningitis, pneumonia, bacteremia, endocarditis, peritonitis, gastroenteritis, and diarrhea. Some cases also highlight the association of this bacterium with human immunodeficiency virus disease [84–87].

*Arcobacter* was the second most abundant genus in the catchment, with 3.796%, showing a moderate decrease to 2.345% in the downstream and a mere 0.001% in the upstream (Fig. 4). *Arcobacter*, a genus associated with pollution and potential pathogenicity, is more abundant in the polluted catchment area and is an alternative fecal- and sewage-associated pollution indicator [88]. Studies in rivers like the Bagmati River in Nepal and the Wonderfonteinspruit catchment in South Africa have shown that *Arcobacter* thrives in environments contaminated with heavy metals and other anthropogenic pollutants, which create favorable conditions for its growth [82, 89]. Researchers have found the presence of genes like intl1 in *Arcobacter*, which have been linked with heavy metals [90, 91]. Although *Arcobacter* is less susceptible to contaminants like Hg, Ag, and Cr, it has also shown high resistance to metals like Mo, Mn, Ni, Co, Pb, and Fe [92]. Metals coupled with organic matter and low oxygen levels enrich *Arcobacter* populations in polluted catchment areas [93]. *Arcobacter* is an emerging food-borne pathogen that poses a growing public health concern due to its ability to cause a variety of human diseases. Infections with *Arcobacter* can lead to gastrointestinal issues, including acute or chronic watery diarrhea, abdominal cramps, and nausea. In more severe cases, it can cause bacteremia, where the bacteria enter the bloodstream, as well as endocarditis, which affects the heart valves, and peritonitis, an inflammation of the lining of the abdomen [94, 95]. Earlier reports of *Arcobacters* in livestock farm effluents stated their ability to cause waterborne disease outbreaks and adaptability to diverse environments, suggesting they can be a contamination source of river water at Kathajodi, which receives the wastewater from potential veterinary and animal rearing activities at its catchment [96]. Downstream sections often have diluted contaminants due to the larger volume of water, making the environment less conducive for *Arcobacter* [97]. The absence of *Arcobacter* in pristine upstream areas indicates its sensitivity to lower pollution levels and lack of specific environmental conditions necessary for its survival. Similar patterns have been observed in the Ganga River in India, where polluted drains significantly contribute to bacterial community changes [73].

*Bacteroides*, while being the third most common genus in the catchment at 3.637%, and 0.128% in downstream, experienced a reduction in abundance, at an almost negligible 0.009% in the upstream area (Fig. 4). *Bacteroides*, primarily fecal indicator bacteria, are more abundant in polluted catchment areas of rivers due to significant fecal contamination and are generally associated with WWTP [82]. The presence of metals like Hg, As, Ni, CO, Pb, Cd, and Cr may favor their growth, and this genus is tolerant to Fe and Zn [73, 98–102]. Research in the Bagmati River, Nepal, and the Wonderfonteinspruit catchment, South Africa, indicates that anthropogenic activities, including industrial discharge and urban runoff, significantly influence *Bacteroides* distribution by providing environments rich in organic matter and pollutants [82, 89]. These microorganisms can cause a variety of serious diseases, some potentially life-threatening. These include conditions like noma (cancrum oris), infections of the tooth’s root (human apical periodontitis), heart valve infections (endocarditis), pelvic inflammatory disease, blood clots with pus (suppurative thrombophlebitis), and infections in wounds [103, 104]. Upstream, where pollution is minimal, *Bacteroides* are scarce, reflecting lower fecal contamination and cleaner water conditions [105]. Downstream, despite persistent contamination, the bacteria are less abundant due to dilution and increased water flow [106]. The ability of pathogenic *Bacteroides* (3.88%) to thrive in anaerobic conditions and their presence in human and animal feces [107], their potentiality to survive in extreme conditions of gut microflora [108] and in aero-tolerant anaerobes environment of oral cavities [109] trace their potential origin to either domestic wastewater generated from the inhabiting population or untreated effluent wastewater of health care centers around the river Kathajodi, as the city is the medical hub of the state. This insight into microbial dynamics within the catchment area underscores the impact of human activities and environmental factors prevailing in the wastewater-receiving river. Similarly, research in the SAR watershed near Los Angeles, CA, USA, reported a high abundance of *Bacteroidetes* originating from agricultural runoff [148].

In the downstream region, the top three bacterial genera were *Aeromonas*, *Acidovorax*, and *Acidimicrobium* (Table S3F). *Aeromonas* had the highest abundance at 4.360% in the downstream area. This genus’s presence increased significantly, where it constituted a mere 0.070% in the upstream area, and it increased further to 1.360% in the catchment (Fig. 4). *Aeromonas* is most abundant in polluted downstream areas of rivers due to higher levels of organic matter and nutrients from human and animal sources, which create an ideal environment for its proliferation [110]. Research reveals that these bacteria have developed efflux pumps, making them tolerant to metals like Zn, Cd, and CO [111]. The development of iron acquisition mechanisms may be another reason for its abundance downstream because iron at specific concentrations helps *Aeromonas* in signaling, metabolism, and development of infections [112]. Apart from this, previous research has confirmed *Aeromonas* showing resistance to metals like As, Hg, Co, Zn, Cd, and Cr [113]. *Aeromonas* bacteria, which naturally thrive in aquatic environments, have been recognized as significant pathogens capable of infecting humans. These bacteria can cause a range of illnesses, from mild to severe. Infections can lead to gastrointestinal issues such as watery diarrhea, dysentery, or even severe, cholera-like diarrhea. Beyond the gut, *Aeromonas* can cause serious complications including sepsis, wound infections, cellulitis, pneumonia, meningitis, and urinary tract infections, especially in vulnerable individuals like those with weakened immune systems. In some cases, infections can escalate, affecting various organs and potentially leading to life-threatening conditions like hemolytic syndrome, kidney disease, or severe skin and soft tissue infections [104, 114, 115]. In pristine upstream areas, the minimal presence of pollutants and lower levels of organic matter contribute to negligible *Aeromonas* populations [116]. Similar patterns are observed in other river systems, such as the Ganga River in India, where varying levels of contamination influence bacterial community structures [73], whereas downstream of the river behind the city, where there are fewer anthropogenic activities and where the pollution from the city is expected to be further diluted in the river stream, showed higher abundances of *Aeromonas*; a notable portion of this bacteria is resilient against water treatment methods. Its presence in drinking water raises concerns, warranting regulatory attention. Due to the widespread occurrence of *Aeromonas* in drinking water and its propensity to develop novel resistance mechanisms, coupled with the presence of various virulence factors, regulatory agencies such as the Environmental Protection Agency have classified *Aeromonas* as part of the “Contaminant Candidate List.” Moreover, the third edition of the World Health Organization’s Guidelines for Drinking-Water Quality has identified *Aeromonas* as a significant concern, emphasizing its potential impact on water safety [117]. This may be because of the contaminants from the catchment area affecting the downstream of the river.

*Acidovorax* was the second most abundant genus in the downstream compared to the other two sites. With a mere 0.016% in upstream, it showed an increase to 0.185% in the catchment and further increased to 0.513% in downstream (Fig. 4). *Acidovorax*, a genus within the *Betaproteobacteria* class, typically inhabits aquatic environments, soil, and plant-associated ecosystems [118]. This bacterium is known for its ability to degrade various organic pollutants, thrive in metal-contaminated environments, oxidize iron, and is resistant to its toxicity [119–121]. The abundance may be due to the presence of Cu and Pb, similar to the findings at the Rophib mining site [122]. In the highly polluted catchment area, its presence was moderate, likely due to the abundance of *Flavobacterium*. Studies conducted in downstream of a wastewater treatment plant in the Iskar River in Bulgaria have shown comparable patterns, where *Acidovorax* was more abundant in polluted downstream areas due to less abundance of *Flavobacterium* [123]. The negligible abundance upstream can be attributed to the cleaner environment with lower nutrient levels and the presence of metals like Cr and Hg, which may not favor its growth [124]. Anthropogenic activities such as industrial discharges, agricultural runoff, and urban wastewater significantly influence the metal and pollutant levels in these environments, affecting the microbial community structure. Factors such as nutrient availability, organic matter, and metal concentrations play crucial roles in determining the habitat suitability and abundance of *Acidovorax* in different river environments [125, 126]. Diseases associated with *Acidovorax* are still unknown because certain species of *Acidovorax* cannot survive above 30 °C. It is a flagellated plant pathogen but can cause hematological malignancy, fever, and sepsis in immunocompromised individuals; however, the exact mechanism for infection in humans is not known [127].

Though present in a very small number, still the third most abundant genus in the downstream was *Acidimicrobium*, with a 0.321% abundance (Fig. 4). This genus was minimally represented in upstream at 0.108% and was merely 0.002% in the catchment area. *Acidimicrobium* species thrive in acidic and metal-rich environments such as acid mine drainage, hot springs, and geothermal features [128]. Their presence in water may be due to their tolerance to high concentrations of metals like Fe, Mn, Cu, Zn, Ni, and Co [129, 130]. They are also known for their bioleaching activity and reducing metals like Fe and Mn [131–133]. The lower abundance in pristine upstream areas can be attributed to lower concentrations of necessary metals [134]. The extreme absence in highly polluted catchment areas is likely due to high levels of toxic metals like Cd and Pb, which inhibit growth [135]. Factors such as metal concentration, pH levels, and organic matter influence their distribution, with essential metals like Fe and Mn supporting growth, while toxic metals like cadmium and lead inhibit it [136]. Industrial discharges, agricultural runoff, and urban wastewater increase metal and pollutant levels, influencing microbial community structures [137]. Similar patterns have been observed in the Rio Tinto in Spain [138] and the Clark Fork River in Montana, USA [139]. The Unclassified group of *Betaproteobacteria* (54.78%) exhibits significant biodegradation capabilities. These bacteria are generally reported from diverse sources such as organic debris, iced fish, and coastal zones and are expected to hold promising biotechnological potential. Their abundance in the DS region of the river may be attributed to their proficiency in biodegradation, and high iron content might be suited for their proliferation [37, 129]. Similarly, water samples from the urban region of the Jialing River also reported a relatively higher prevalence of Betaproteobacteria [81].

The abundance and diversity of bacterial communities in aquatic ecosystems are influenced by a range of environmental factors, including pH, temperature, dissolved oxygen, and nutrient availability. These factors are further impacted by urbanization-associated processes like damming and water intake, which reduce hydrodynamics and self-purification capacity, disrupting natural ecosystem functions [140]. Pollution gradients, such as nutrient enrichment and heavy metal accumulation, can drastically alter microbial community structure and function by promoting high microbial biomass but causing instability when nutrients become limiting. Heavy metals, in particular, suppress enzymatic activity and metabolic processes in microbial communities [140, 141]. Interactions among microbial genera, such as competition between *Flavobacterium* and *Proteobacteria*, are also influenced by abiotic factors like pH and nutrient availability, with higher pH favoring these groups [140].

Deterministic factors like pH, salinity, and stochastic processes play a role in shaping microbial shifts in regions like the US, CM, and DS. In particular, nutrient and metal accumulation in sediments result in uneven bacterial responses, leading to a restructuring of microbial communities. Studies show that nutrient enrichment promotes microbial productivity, while the toxic effects of heavy metals inhibit enzymatic pathways, with their interaction explaining the microbial community variance [142]. These changes significantly affect key ecosystem functions such as biogeochemical cycling, oxygen dynamics, and pathogen suppression, with a reduction in microbial diversity posing a threat to ecosystem stability and resilience [143].

**Fig. 5:**
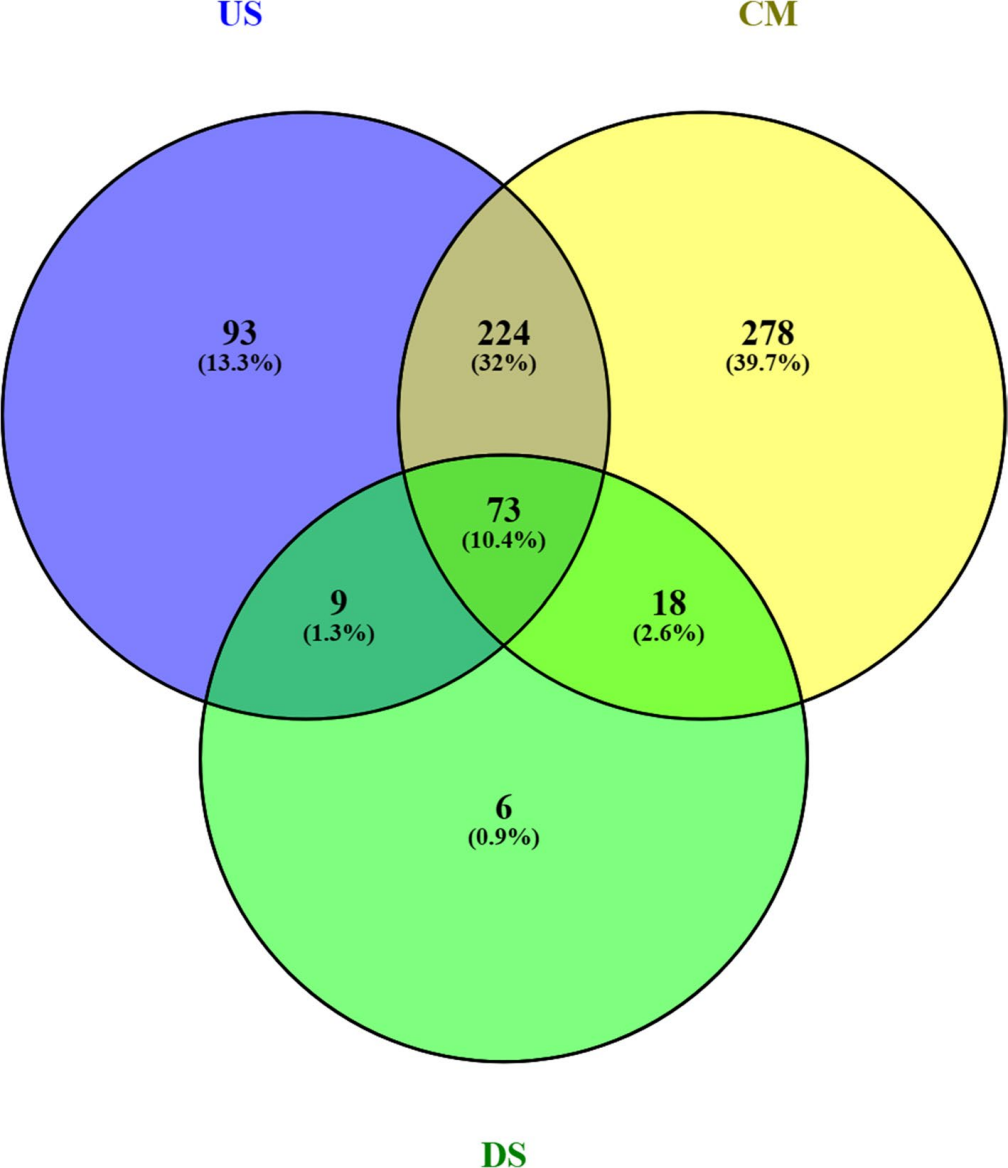
Venn diagram at genera level representing the number of unique and shared OTUs with the samples

Additionally, the highest number of unclassified bacteria at the genera level was observed in the US (37.081%), followed by CM (34.482%) and DS (24.879%) (Table S3F). From the Venn diagram, 93, 278, and 6 unique genera were found in all three sites US, CM, and DS, respectively, while 73 genera were common to all (Fig. 5). Detection of the extremely high number of unique genera could be attributed to the prevailing distinctively different environments of the river at that location due to inadvertently accumulated wastes received from a range of anthropogenic events.

### Predictive functional profiling of microbial communities

**Fig. 6:**
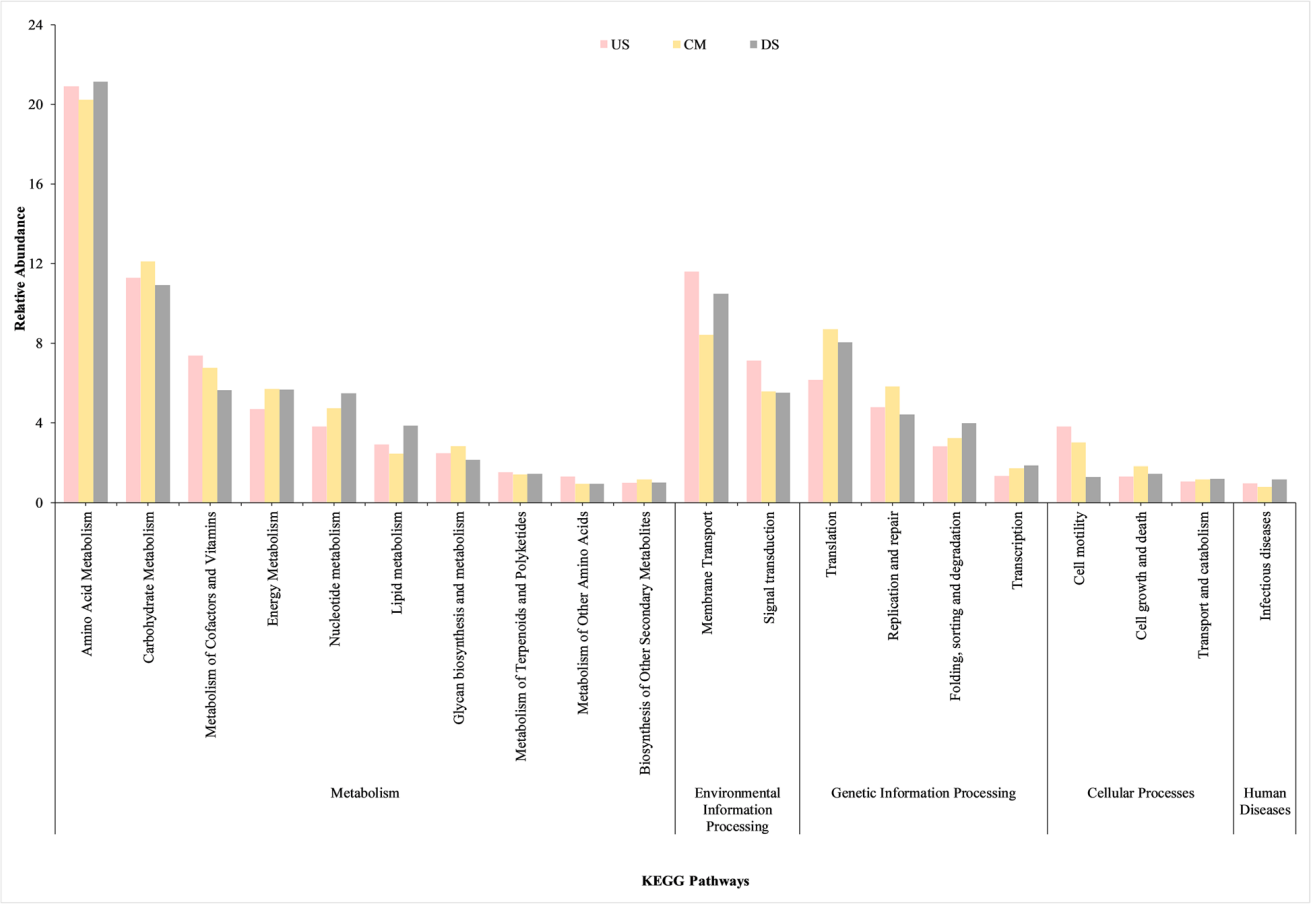
The bar graph illustrates a comparison of the top 20 relative abundances (%) of predicted microbial KEGG annotations for unigenes at levels 1 and 2 in three distinct regions of the Kathajodi River, as identified by the SEED database. The *x*-axis of the graph denotes the KEGG pathways, while the *y*-axis indicates the relative abundance of each respective pathway

Functional profiles of microbiota were determined for three different samples (US, CM, and DS) using individual OTUs through MG-RAST analysis. The results unveiled 6 distinct groups at level 1, 53 at level 2, and 267 at level 3 of KEGG Orthology (Table S4), along with 25 functional categories according to COG classification (Table S5). These categories included functions like signal transduction, membrane transport, cellular community among prokaryotes, energy metabolism, and carbohydrate metabolism of KEGG Orthology. Notably, metabolism of cofactors and vitamins (7.35%), membrane transport (11.59%), signal transduction (7.10%), and cell motility (3.82%) were particularly abundant in the US samples. Meanwhile, carbohydrate metabolism (12.11%), glycan biosynthesis and metabolism (2.83%), translation (8.68%), and replication and repair (5.81%) were more prevalent in the CM samples. In contrast, the DS samples exhibited higher levels of amino acid metabolism (21.12%), nucleotide metabolism (5.47%), lipid metabolism (3.85%), and folding, sorting, and degradation (3.99%) (Fig. 6). These metabolic pathways primarily serve the fundamental purpose of providing energy and essential building blocks required for synthesizing biological molecules. This is crucial for sustaining the life processes of microorganisms and ensuring the maintenance of metabolic equilibrium within the ecosystem [144]. The analysis of metabolic pathways at the KEGG level 2 of the Guixi River indicated that certain pathways, namely carbohydrate metabolism and energy metabolism, exhibited the highest functional importance. These metabolic pathways primarily serve the fundamental purpose of providing energy and essential building blocks required for synthesizing biological molecules. Another study conducted in the Tama River watershed, Japan, affected by human activities, revealed that 28% of second-tier KO categories focused on enzyme families, with over 8% related to carbohydrate metabolism, more than 7% to translation, over 6% to energy, and 5% to replication and repair [145]. Similarly, research conducted in sediment from three different polluted sites along the Ganga River at Kanpur found that genes associated with carbohydrate metabolism were prevalent, accounting for 11.26%, 10.53%, and 9.73% of the identified genes [146].

**Fig. 7:**
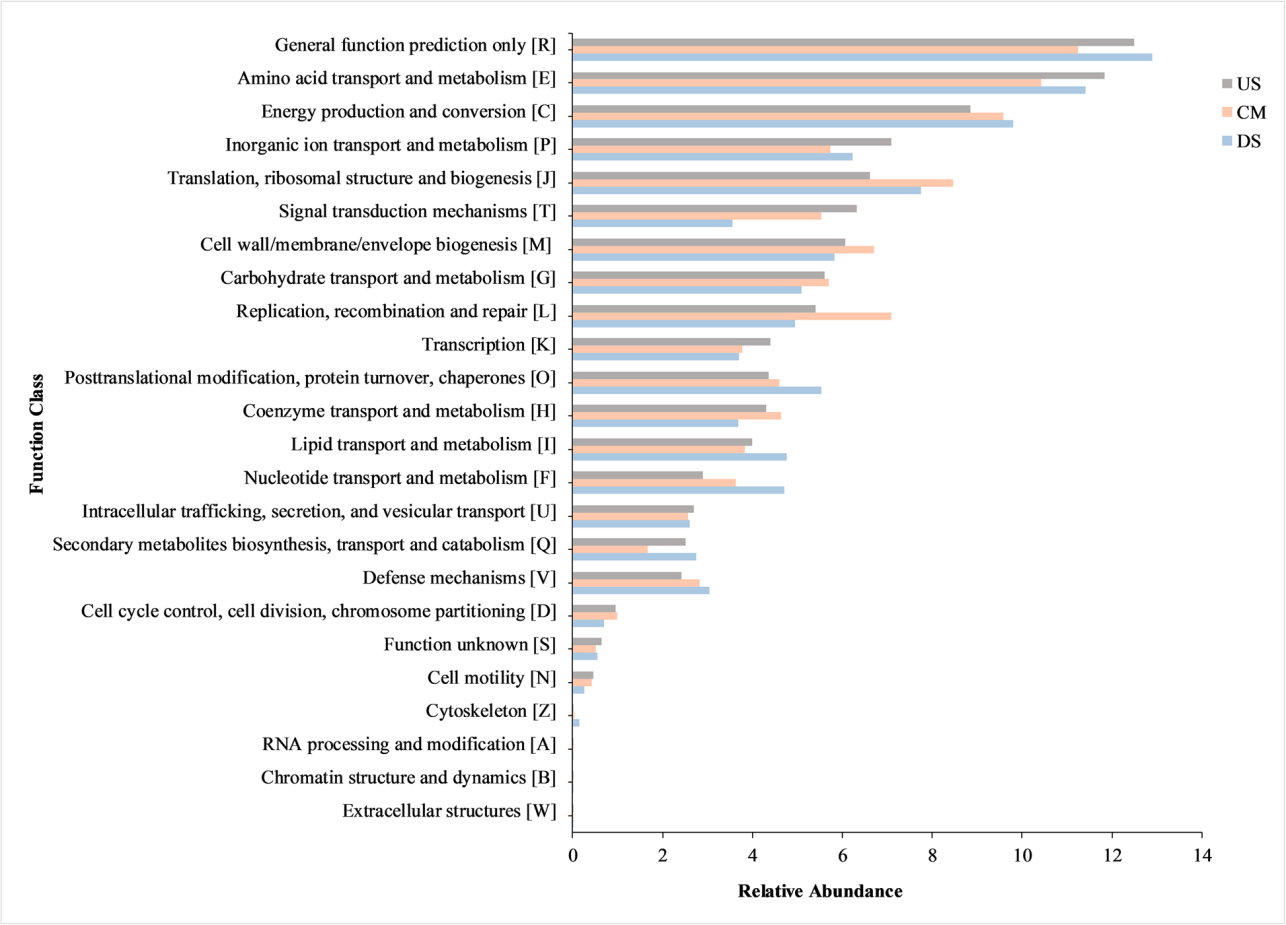
The bar graph depicts the distribution of metagenome reads assigned to 25 COG functional categories as obtained through MG-Rast. The X-axis illustrates the relative abundance (%) of COG functions, while the y-axis displays the selected COG functional terms

The distribution of COG functions indicated distinct patterns in different sample locations. In the US sample, there was a notable presence of functions related to amino acid transport and metabolism (E) (11.827%), inorganic ion transport and metabolism (P) (7.091%), transcription (K) (4.397%), and signal transduction mechanisms (T) (6.323%) (Fig. 7). On the other hand, the catchment area (CM) showed higher proportions of translation, ribosomal structure, and biogenesis (J) (8.464%), cell wall/membrane/envelope biogenesis (M) (6.697%), and carbohydrate transport and metabolism (G) (5.701%). Lastly, the DS sample displayed a prevalence of functions such as general function prediction only (R) (12.890%), energy production and conversion (C) (9.795%), and posttranslational modification, protein turnover, and chaperones (O) (5.532%) (Table S4). A research study conducted in New Delhi’s polluted stretch of the Yamuna River unveiled the prevalence of various genes associated with functions like general function prediction, translation, ribosomal structure, and carbohydrate metabolism, which were prominent at COG function level 2. Additionally, many genes were related to replication, recombination, and repair. Furthermore, the analysis showed that over 2.68% of genes in all samples were dedicated to defense mechanisms against pollution [147]. In a separate study in Kanpur, the distribution of genes in the sediment of three polluted sites along the River Ganga was analyzed. Approximately, 12.00%, 11.67%, and 13.33% of genes were linked to general function prediction. Moreover, a higher proportion of genes associated with translation, ribosomal structure, and biogenesis were observed across all three sediment metagenome datasets [146]. Prediction of the above functional potentials at the pollution sites of the above rivers corroborates our findings at the catchment area of Kathajodi, which has been found to be contaminated with profuse anthropogenic activity (Fig. 7).

### Canonical correspondence analysis of microbial communities

**Fig. 8:**
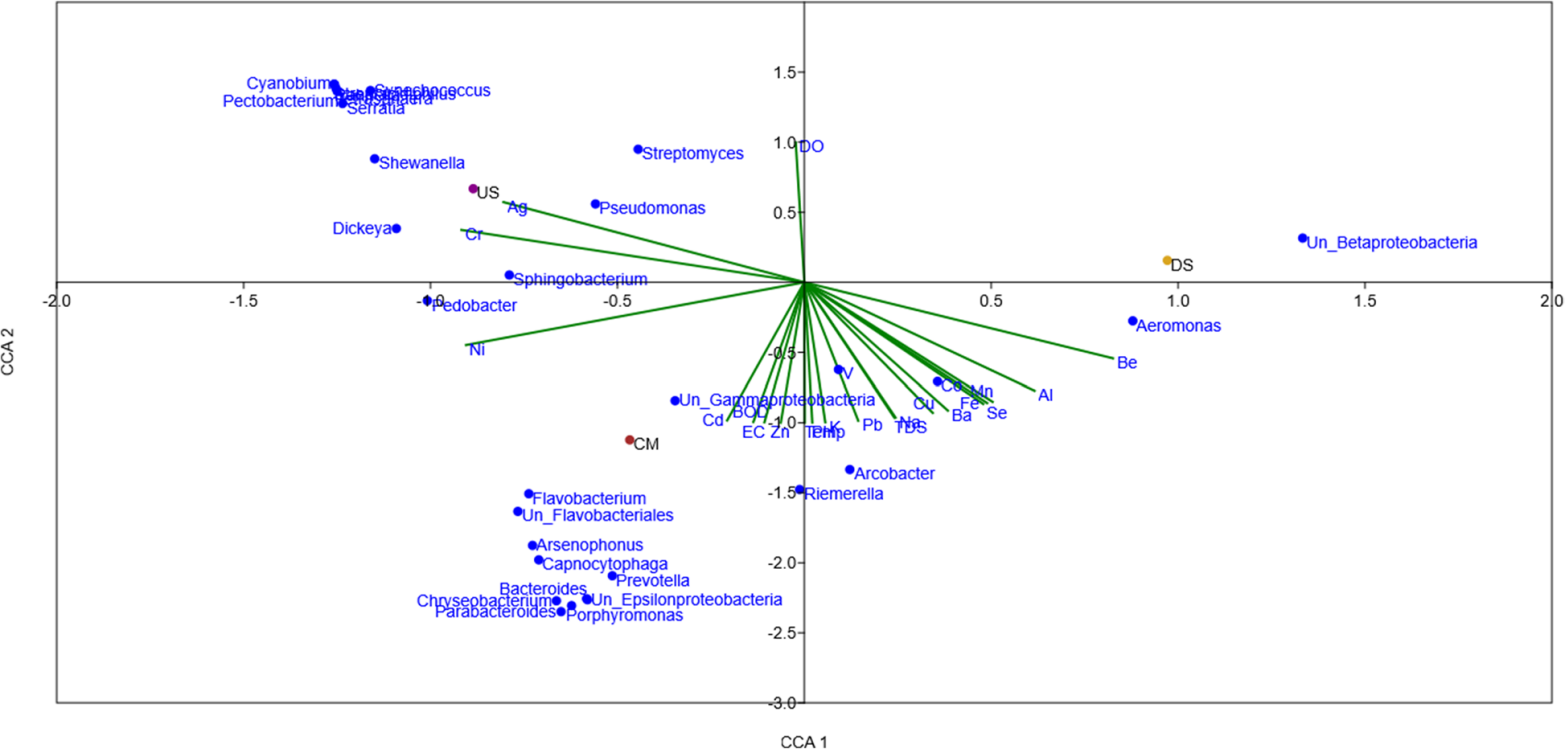
Canonical correspondence analysis (CCA) biplot showing the relationships between microbial taxa (blue dots), environmental parameters (green vectors), and sampling sites (US, CM, DS) across the Kathajodi River

The canonical correspondence analysis (CCA) ordination provided a clear visualization of microbial community structuring in relation to physicochemical parameters across the Kathajodi River (Fig. 8). The ordination separated the US, CM, and DS samples, indicating that distinct environmental drivers operate at each site.

In the US section, bacterial taxa such as *Pseudomonas* and *Shewanella* clustered close to trace metals like Ag and Cr. These genera are typically metabolically versatile and thrive in oxygen-rich, nutrient-variable conditions, supporting oxidative metabolism and biogeochemical cycling [3].

Conversely, CM samples grouped near *Flavobacterium* and *Bacteroides* taxa clustered close to trace metals like Cd, BOD, and Zn, commonly associated with sewage and organic-rich effluents. The clustering reflects the effect of anthropogenic inputs, including municipal drainage and agricultural runoff, which stimulate carbohydrate-degrading consortia [4]. This pattern aligns with KEGG-based functional predictions showing enrichment in carbohydrate metabolism, glycan biosynthesis, and translation pathways at CM (Fig. 6). COG profiles (Fig. 7) corroborated these findings, with a higher proportion of translation (J) and cell wall/membrane biogenesis (M) functions, indicative of copiotrophic growth under nutrient-rich conditions [17, 145].

The DS site, however, did not show direct alignment with measured physicochemical parameters, suggesting that its community structure is influenced by unmeasured or cumulative stressors rather than single chemical drivers. Instead, the right-lower ordination quadrant revealed associations of genera such as *Aeromonas*, *Arcobacter*, and unclassified *Betaproteobacteria*, which clustered close to trace metals like K, Pb, Na, TDS, Cu, Co, Mn, Al, and Fe. These taxa are known for their tolerance to heavy metals, biofilm formation, and, in some cases, opportunistic pathogenicity—traits that enable persistence under polluted and chemically stressed conditions [6]. Functional profiling revealed DS communities to be enriched in amino acid and nucleotide metabolism, as well as protein-folding pathways (Fig. 6), which are characteristic of stress response and homeostasis mechanisms under chemical pressure [1].

Such site-specific divergence is consistent with findings from other urban rivers, including the Ganga [146], Yamuna [147], and the Tama River, Japan [145], suggesting that local pollution regimes strongly influence microbial community assembly and functional potential.

## Conclusion

The metagenomic analysis of the Kathajodi River catchment area revealed significant insights into the shift in microbial diversity and the functional relevance, as influenced by the anthropogenic activities of the urban city around. The pristine upstream region harbored beneficial genera such as *Shewanella*, *Pseudomonas*, and *Synechococcus*, while the polluted catchment was enriched with pollution and fecal indicators including *Flavobacterium*, *Arcobacter*, and *Bacteroides*. Downstream, the community composition shifted again, with genera such as *Aeromonas* and *Acidovorax* indicating residual pollution and adaptation to metal-rich conditions. The dominance of *Bacteroidetes* in the catchment area underscored the impact of urbanization and wastewater discharge on the microbial community structure. The observed variations in genera abundance and identification of unique genera across the three sites highlighted the distinct environmental conditions prevailing there. Additionally, the predictive functional profiling provided valuable information on the metabolic capabilities of the microbial communities, with specific functions associated with different sites, reflecting adaptations to local environmental conditions. The clear separation of sites in CCA ordination highlights that dissolved oxygen and heavy metal pollution are dominant forces shaping community assembly. However, the study is limited by single-time sampling, uneven site coverage, and the exclusion of sediments, which may overlook finer spatial variations in microbial diversity. Future work should include multi-seasonal and sediment analyses. Additionally, key aspects such as identifying microbial genes involved in pollutant degradation, antimicrobial resistance gene distribution, and horizontal gene transfer mechanisms remain unexplored. Addressing these gaps in future studies could provide deeper insights into microbial responses to pollution and their potential role in bioremediation. These findings contribute to a broader understanding of how human activities shape riverine microbial ecosystems and emphasize the importance of monitoring microbial indicators alongside physicochemical parameters to assess river health and manage anthropogenic impacts on water quality. Further research and targeted interventions are essential for mitigating the adverse effects of urbanization on aquatic ecosystems and microbial risks associated with untreated discharges, ensuring the sustainable management of surface water resources.

## Supplementary Information

Supplementary Material 1. Table S1. Physicochemical Parameters and Heavy Metal Analysis. Table S2. Metagenome sequence and assembly statistics. Table S3 (A). Microbial community composition at domain level. Table S3 (B). Microbial community composition at phylum level. Table S3 (F). Microbial community composition at genus level. Table S4. Prediction of functional groups of OTUs among all the samples by TaxfFun. Table S5. Prediction of COG functional classification by SEED database.

## Data Availability

The raw metagenomic sequence data that support the findings of this study have been submitted to the NCBI's Sequence Read Archive (SRA) database under the BioProject (PRJNA1008397), with accession numbers SRR25894123 (DS), SRR25894124 (CM), and SRR25894125 (US). The data described in the article can be freely and openly accessed on the figshare depository under https://doi.org/10.6084/m9.figshare.9197972.v1, (Data set 1 https://figshare.com/articles/dataset/Data_set_1/9197972/1).

## Abbreviations

BOD: Biochemical oxygen demand
CM: Catchment area
DS: Downstream
EC: Electrical conductivity
HGT: Horizontal gene transfer
TDS: Total dissolved solids
US: Upstream
KEGG: Kyoto Encyclopaedia of Genes and Genomes
COG: Clusters of Orthologous Groups
