# Supplementary material for: Metagenomic analysis of microbiome spatial dynamics in urban river confluence affected by city wastewater
Source: Genomics Inform. 2025 Dec 4;23:27. doi: 10.1186/s44342-025-00054-3 (PMC12676890; doi:10.1186/s44342-025-00054-3)
Supplement: Supplementary file 1 — Supplementary Material 1. Table S1. Physicochemical Parameters and Heavy Metal Analysis. Table S2. Metagenome sequence and assembly statistics. Table S3 (A). Microbial community composition at domain level. Table S3 (B). Microbial community composition at phylum level. Table S3 (F). Microbial community composition at genus level. Table S4. Prediction of functional groups of OTUs among all the samples by TaxfFun. Table S5. Prediction of COG functional classification by SEED database. [file 44342_2025_54_MOESM1_ESM.zip › Supplementary/Table_S2.docx]

| **Category** | **US** | **CM** | **DS** |
| --- | --- | --- | --- |
| **Sequencing method/platform** | Illumina HiSeq X10 | Illumina HiSeq X10 | Illumina HiSeq X10 |
| **Library type** | Paired end read (150bp x 2) | Paired end read (150bp x 2) | Paired end read (150bp x 2) |
| **Project Type** | De novo whole genome metagenomics | De novo whole genome metagenomics | De novo whole genome metagenomics |
| **N50** | 2,099 | 1,467 | 1,234 |
| **Total number of reads** | **R1:** 21,283,221 | **R1:** 25,302,269 | **R1:** 37,221,935 |
|  | **R2:** 21,283,221 | **R2:** 25,302,269 | **R2:** 37,221,935 |
| **Total sequence (bp)** | 4,00,70,768 | 4,65,72,024 | 6,85,86,460 |
| **QC mean reads length (bp)** | 155 ± 29 bp | 157 ± 31 bp | 157 ± 31 bp |
| **Total Identified rRNA Features** | 14,865 | 26,002 | 37,784 |
| **Mean GC percent** | 56 ± 10 % | 47 ± 14 % | 53 ± 14 % |
| **Total OTUs** | 18760 | 13179 | 110230 |
| **Phylum** | 25 | 26 | 14 |
| **Class** | 42 | 49 | 24 |
| **Order** | 87 | 105 | 53 |
| **Family** | 173 | 226 | 79 |
| **Genus** | 399 | 593 | 106 |
| **Species** | 881 | 1475 | 160 |

**Supplementary Table S2:** **Metagenome sequence and assembly statistics**
